# Capsule endoscopy aspiration and respiratory physician’s treatment insights: a case report and literature review

**DOI:** 10.3389/fmed.2024.1442245

**Published:** 2024-11-28

**Authors:** Yinxue Zhou, Hongmei Wang, Min Zhuang, Hua Liu, Lijie Qi, Lingyun Zhang, Jiaxing Sun

**Affiliations:** ^1^Department of Respiratory and Critical Care Medicine, The Affiliated Hospital of Qingdao University, Qingdao, China; ^2^Department of Gastroenterology, The Affiliated Hospital of Qingdao University, Qingdao, China; ^3^Department of Anesthesiology, The Affiliated Hospital of Qingdao University, Qingdao, China

**Keywords:** capsule endoscope, complication, aspiration, bronchoscope, case report

## Abstract

**Background:**

Capsule endoscopy (CE) is widely used for intestinal examination; however, capsule aspiration into the airway is a serious complication that requires urgent intervention. We present a management case report and review 39 cases from 2003 to 2023, providing insights into the prevention and treatment of capsule aspiration.

**Case presentation:**

A 69-year-old man with chronic bronchitis and emphysema presented with 7 months of intermittent melena. After swallowing a capsule endoscope (PillCam^™^ SB 3), he had a brief cough and chest tightness. Imaging confirmed aspiration in the right intermediate bronchus, and non-invasive removal procedures were unsuccessful.

**Methods:**

Real-time imaging confirmed the lodged capsule. Non-invasive methods, such as coughing and chest percussion, were unsuccessful. Therefore, flexible bronchoscopy was performed under general anesthesia to retrieve the capsule using a snare, which was then placed into the duodenum using a gastroscope.

**Results:**

The capsule was successfully retrieved, and the patient recovered well, completing the endoscopy without further issues.

**Conclusion:**

Our case study and literature review highlight the need for careful attention to high-risk groups in CE, including the elderly and individuals with neurological or swallowing difficulties. A thorough history review and real-time monitoring are essential for preventing complications. Bronchoscopy is preferred for CE retrieval due to its advantages. Manufacturers are urged to improve CE safety, with respiratory physicians helping internists in managing this potentially life-threatening complication.

## Introduction

Capsule endoscopy (CE) is a primary diagnostic tool for small bowel diseases due to its non-invasiveness and safety in diagnosing mucosal diseases. It has pioneered a new mode of examination for the entire digestive tract (1, 2). This innovative technique involves swallowing a small, pill-sized capsule containing a camera that captures images as it traverses the digestive tract. Capsule endoscopy is very useful for investigating obscure gastrointestinal bleeding (3), diagnosing Crohn’s disease (4), assessing small bowel disorders (5), and detecting abnormalities, such as polyps or tumors in the small intestine (6). Its non-invasive nature makes it a valuable tool for patients who may find traditional endoscopic procedures uncomfortable or challenging. In addition, capsule endoscopy is an effective diagnostic tool when conventional imaging methods fail to identify the underlying causes of gastrointestinal symptoms. However, adverse events such as CE retention, difficulty swallowing, aspiration, and missed diagnosis can still occur in clinical applications (1).

Despite occasional instances in previously documented cases where patients were able to naturally expel aspirated capsules from the respiratory tract and later re-ingest them into the digestive system (7), capsule aspiration remains a serious complication requiring prompt intervention. While spontaneous resolution is possible in some cases, the unpredictable nature of this response underscores the need for vigilance and intervention to mitigate potential adverse outcomes. Therefore, clinicians must remain alert to the severity of capsule aspiration as a serious complication, emphasizing the need for timely and appropriate medical management. CE aspiration is an emergent complication that requires immediate intervention. Previous studies have reported several methods for managing CE aspiration, including the use of rigid or flexible bronchoscopy with various tools to aid in manipulation (8). We further conducted a detailed analysis of previous studies documenting cases of CE aspiration to identify common clinical features and effective management strategies to provide valuable references for future instances.

However, as of now, standardized treatment strategies for this potentially fatal complication have not been established. This report examines the causes of capsule aspiration and suggests preventive measures, emphasizing the importance of physicians familiarizing themselves with management options.

## Case presentation

**Figure 1:**
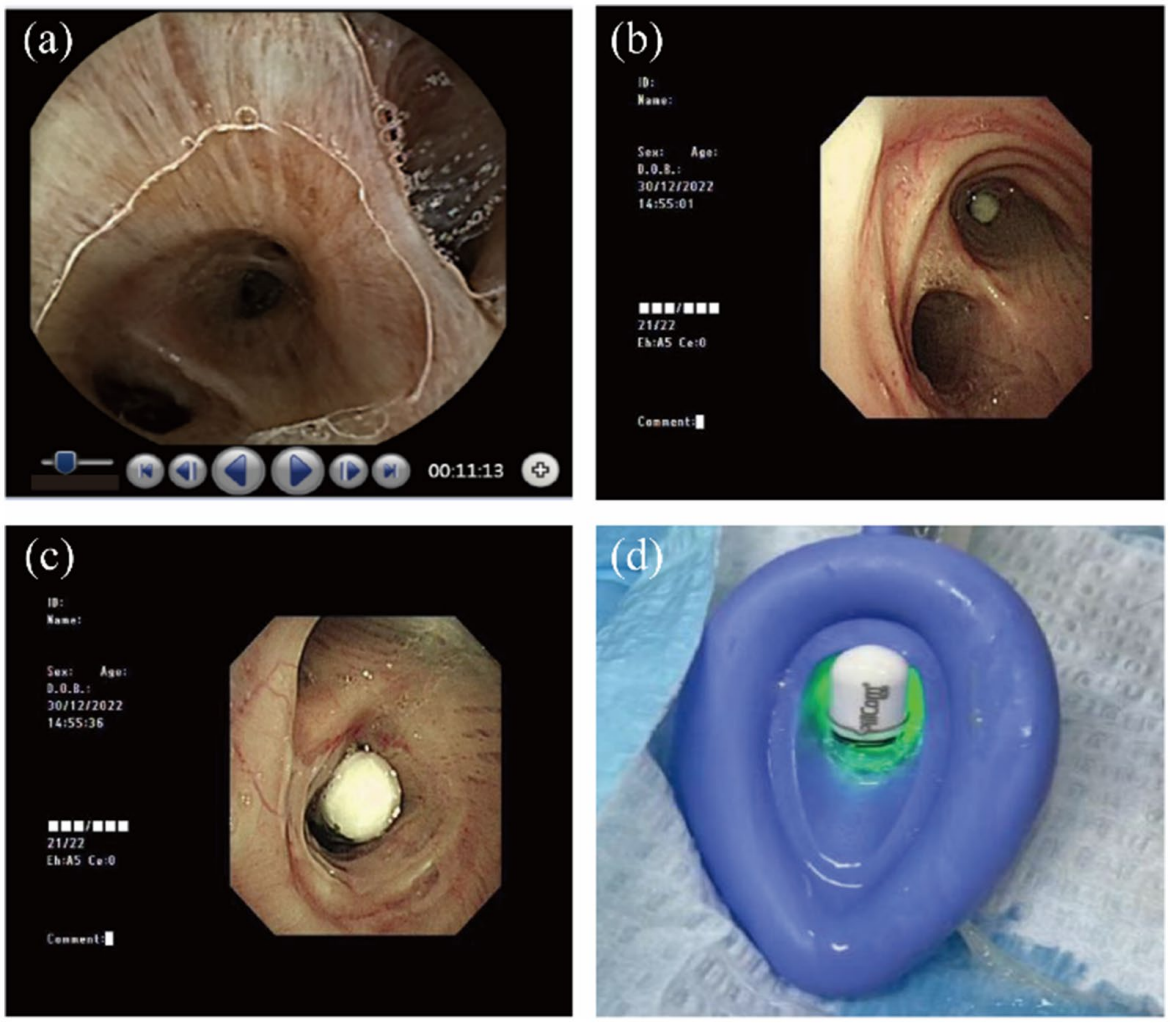
Removal process of the capsule endoscope **(a)** real-time capsule endoscopic images showing the tracheal cartilage ring and typical airways. **(b,c)** Bronchoscopic view shows the capsule lodged in the bronchus intermedius. **(b)** Tracheal carina and **(c)** right main bronchus. **(d)** Capsule endoscopy successfully removed the capsule from the airway.

A 69-year-old man with a history of chronic bronchitis and emphysema presented to our hospital with intermittent melena for 7 months. Previous endoscopy revealed chronic atrophic gastritis and multiple colon polyposis. Upon swallowing a capsule endoscope (PillCam^™^ SB 3 Capsule Endoscopy System, Medtronic), the patient immediately experienced coughing and chest tightness lasting approximately 1 min. The physical examination revealed decreased inspiratory sounds and expiratory wheezing in the central part of the right lung, indicating capsule aspiration. Meanwhile, the CE image showed a real-time recording of the tracheal cartilage ring and typical airways, supporting this diagnosis (Figure 1a). As the capsule was lodged in the right intermediate bronchus, the symptoms of cough and dysphagia were not evident. We encouraged the patient to cough actively and performed postural drainage and chest percussion therapy in an attempt to remove the capsule spontaneously. Unfortunately, the capsule remained lodged in the right intermediate bronchus.

As previously reported, flexible bronchoscopy or hard endoscopy examination is the most preferred approach to retrieve the capsule. Given the patient’s history of pulmonary disease and the unpredictable timing of the surgery, we opted to perform the procedure with general anesthesia, using a laryngeal mask airway for ventilation support. The bronchoscopy revealed that the capsule was located in the right intermediate bronchus (Figures 1b,c). Despite the challenge posed by the capsule’s smooth surface coated with mucus, we were able to successfully retrieve it with the use of a snare (Figure 1d).

**Figure 2:**
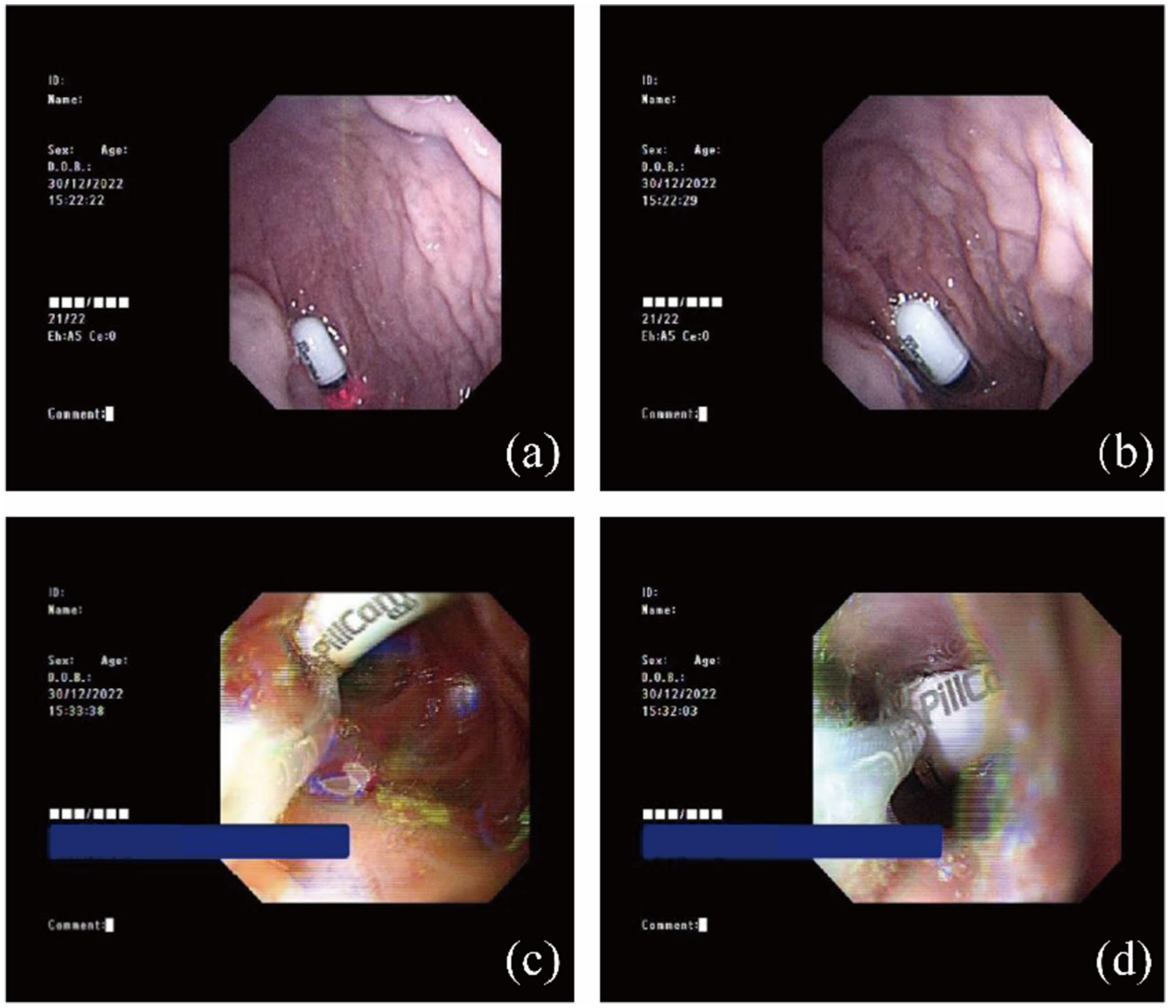
Successfully pushed the capsule endoscope into the duodenum. **(a,b)** The capsule endoscope in the stomach. **(c,d)** The capsule endoscope in the duodenum.

Given the patient’s high risk of aspiration, we decided to bypass the esophagus and directly place the capsule into the duodenum using a gastroscope. However, during the procedure, we encountered several obstacles, such as difficulty in accurately entering the esophagus with the gastroscope and the capsule falling back into the trachea. This occurred because the capsule in front of the gastroscope obstructed the endoscope’s view of its progression. Fortunately, by adjusting the patient’s position and modifying the method of securing the capsule to the gastroscope, we successfully implanted the capsule into the duodenum (Figure 2). The patient recovered well and completed the video capsule endoscopy the following day.

## Discussion and literature review

CE, as an effective tool for directly evaluating the intestinal lumen via a video, has become widely accepted in recent clinical gastrointestinal investigations. With high patient acceptance and safety, the indications for CE have expanded to include obscure gastrointestinal bleeding, inflammatory bowel diseases without ileus, and other abnormalities that cannot be confirmed by gastrointestinal endoscopy or colonoscopy (9, 10). The overall complication rate of CE is low, with CE retention occurring in approximately 2% of all cases (11). However, one isolated but emergent complication that requires multi-departmental effort is capsule aspiration into the trachea or bronchial tree. Schneider et al. published the first case of M2A capsule aspiration since the diagnostic method became available in general clinical practice in 2003. The patient aspirated the capsule into the right main bronchus on the fourth attempt, but coughed it out after 2 min. The subsequent evaluation confirmed appropriate swallowing and small-bowel visualization (12).

While the case had a relatively benign outcome, a broader review of the literature revealed a more varied and complex picture of capsule aspiration events. Using “capsule endoscopy” and “aspiration” as keywords, a search on PubMed identified a total of 39 cases reported between 2003 and 2023 (Table 1). The majority of the patients were over 60 years old (38/39, 97.4%), with most being male (36/39, 92.3%). All patients had underlying conditions; however, only three cases (7.7%) had a documented history of swallowing difficulties. One-third of the patients (13/39) experienced difficulties swallowing the capsule. After aspiration, except for a few patients (8/39, 20.5%) who remained asymptomatic, the majority of them experienced coughing, a sensation of a foreign body in the throat, and respiratory difficulties. The capsules mostly lodged into the right main bronchus. Following aspiration, a subset of patients (18/39) either spontaneously coughed out the capsule or re-swallowed it into their digestive tract. Bronchoscopy has emerged as the most commonly used method for managing capsule endoscopy aspiration, and in this case, the capsule was successfully retrieved using a flexible bronchoscope. Notably, in one case involving a 77-year-old man, despite briefly expelling the capsule after aspiration, the patient succumbed to a cerebrovascular accident. In addition, another patient suffered atelectasis following capsule retrieval, while the remaining patients had good post-extraction outcomes. A logistic regression analysis was conducted to assess whether factors such as age, sex, and the presence of coughing symptoms had a significant effect on poor prognosis. The results indicated that these variables did not have any statistically significant impact (*p* > 0.05, Table 2). The small sample size of 39 cases might have limited the ability to detect meaningful associations. Patient variability, such as comorbidities and symptom severity, might have obscured the impact of the analyzed variables. Unmeasured factors, such as individual responses to aspiration or medical management, could also have played a larger role, making age, sex, and coughing symptoms less predictive. In addition, with only two poor outcomes, it was difficult to identify clear predictors.

**Table 1:** Review of capsule endoscopy aspiration (2003–2023).

Age	Sex	Medical history	Swallowing disorder	Difficulty swallowing the capsule	Symptom	Time duration for which the capsule stayed in the trachea	Location	Mode of extraction	References
64	Male	Anemia and mechanical mitral valve replacement	NS	Yes	Cough	2 min	Right main bronchus	Spontaneous	(12)
74	Male	Anemia and ankylosing spondylitis with the involvement of the cervical spine	No	No	Mild respiratory distress while walking	2 days	Right main bronchus	Flexible bronchoscopy	(19)
69	Female	Anemia	No	No	Cough	50 s	Tracheal bifurcation	Spontaneous	(20)
87	Male	Bladder cancer, congestive heart failure, atrial fibrillation, coronary artery disease, and mild renal insufficiency	NS	Yes	Foreign body sensation	NS	Right main bronchus	Rigid bronchoscopy	(21)
93	Male	No significant past medical history	No	No	Minimal cough	8 h	Bronchus	Spontaneous	(22)
67	Male	Anemia, hypertension, diabetes mellitus, and cerebrovascular accident	Yes	Yes	Cough and dysphonia	Hours	Left main bronchus	Rigid bronchoscope	(23)
75	Male	Anemia	No	Yes	Cough	NS	Right main bronchus	Spontaneous	(24)
65	Male	Sigmoid resection	Yes	Yes	Cough	NS	Right main bronchus	Spontaneous	(18)
90	Male	Ischemic stroke	No	No	No	NS	Bronchus	Rigid bronchoscopy	(25)
76	Male	Anemia	No	No	Weak coughing	15 s	NS	Spontaneous	(26)
85	Male	Anemia	No	Yes	Slight pain	8 h	Bronchus	NS	(27)
75	Male	Cerebrovascular accident	No	No	Cough	NS	Left main bronchus	Bronchoscopy	(28)
90	Male	Coronary artery disease, atrial fibrillation, peripheral vascular disease, cerebrovascular accidents, and chronic obstructive pulmonary disease	NS	No	NS	Hours-days	Left main bronchus	Chest percussive therapy (CPT) with postural drainage and bronchoscopic removal.	(29)
73	Male	Renal cell carcinoma, bovine mitral valve replacement, and hyperlipidemia	No	No	Cough and a sensation of capsule impaction at the back of the throat	NS	Right main bronchus	Bronchoscopy	(29)
80	Male	Advanced Parkinson’s disease	No	Yes	Persistent cough and transitory breathing difficulties	20s	Tracheal bifurcation	NS	(30)
82	Male	Anemia and arterial hypertension.	No	No	No	6 days	Left main bronchus then right	Spontaneous	(8)
65	Male	Ethanol-induced cirrhosis, chronic pancreatitis, and chronic obstructive pulmonary disease	No	No	No	NS	Right main bronchus	Rigid bronchoscopy	(31)
73	Male	COPD and persistent iron deficiency anemia (IDA)	NS	NS	Brief cough	NS	Left main bronchus	Rigid bronchoscopy	(31)
81	Male	Recurrent IDA	NS	NS	Fleeting choking sensation	NS	Right main bronchus	Rigid bronchoscopy	(31)
83	Male	Chronic obstructive pulmonary disease and gastroesophageal reflux disease	No	Yes	Mild shortness of breath	NS	Left main bronchus	Bronchoscopy	(32)
77	Female	Hysterectomy	No	NS	Choking	NS	NS	Spontaneous	(33)
80	Male	Chronic obstructive airway disease	NS	NS	Cough	NS	Right main bronchus-main bronchus	Spontaneous	(34)
88	Male	Anemia	NS	NS	Dyspnea.	2h45min	Tracheal bifurcation	Spontaneous	(34)
69	Male	Gastrointestinal bleeding	NS	NS	Cough	NS	Right main bronchus	Flexible bronchoscopy	(35)
78	Male	Chronic kidney failure	NS	Yes	Vigorous coughing	2 min	Tracheal bifurcation	spontaneous	(36)
78	Male	Previous gastric ulcer	No	Yes	Cough	3 min	Tracheal bifurcation	Spontaneous	(37)
83	Male	Iron-deficiency anemia	Yes	Yes	Regurgitation	8 h	Bronchus	Flexible bronchoscopy	(38)
81	Male	NS	NS	NS	Cough	110 days	Left main bronchus	Flexible bronchoscopy	(39)
92	Male	Parkinson’s disease and diabetes	No	No	Dyspnea and vigorous coughing	4 h	Trachea	Spontaneous	(40)
85	Male	Type 2 diabetes, hypertension, and atrial fibrillation	NS	Yes	Foreign body sensation	220 s	NS	Spontaneous	(41)
82	Male	Anemia	No	No	Self-limited coughing, fever, leukocytosis, and dyspnea	25 min	Tracheal bifurcation	Spontaneous	(42)
74	Male	Ischemic stroke and chronic obstructive pulmonary disease	No	NS	Choking episode with mild respiratory distress, bouts of coughing, and oxygen desaturation.	NS	Left main bronchus	Rigid bronchoscopy	(43)
52	Female	Gastroesophageal reflux disease	NS	NS	Dyspnea.	NS	Right lower lobe bronchus just distal to the bronchus intermedius	Rigid bronchoscopy	(44)
81	Male	Alzheimer’s disease and on antiplatelet drug therapy	No	NS	Cough	17 s	Bronchus	Spontaneous	(45)
75	Male	Anemia	No	No	No	7 h	Trachea	Spontaneous	(46)
84	Male	NS	NS	No	No	10 h	Trachea	Spontaneous	(47)
92	Male	Gout, myelodysplastic syndrome, chronic kidney disease, and iron deficiency anemia	NS	Yes	No	NS	Bronchus	Bronchoscopy	(48)
87	Male	Anemia	NS	No	Intermittent coughing and desaturation, requiring supplemental oxygen	NS	Right main bronchus	Flexible bronchoscopy	(49)
73	Male	Hypertension, atrial fibrillation, liver cirrhosis, chronic obstructive pulmonary disease, rheumatoid arthritis, chronic kidney disease, hemicolectomy, and splenectomy.	No	No	Vigorous coughing and reported chest discomfort.	NS	Left main bronchus	Flexible bronchoscopy	(50)

**Table 2:** Statistical analysis of the demographics and cough symptoms impacting the prognosis after capsule endoscopy retrieval.

Outcome	Percentage	*p*-value	OR	95% CI
Age (≥75)	69.2% (27/39)	0.681	1.042	【0.856, 1.269】
Sex (female)	7.7% (3/39)	0.998	0.004	【0.001, 10.040】
Cough	43.6% (17/39)	0.998	0.498	【0.003, 9.263】
Poor prognosis	5.1% (2/39)	\	\	\

In summary, capsule aspiration primarily occurs in elderly patients, with key risk factors including depressed mental status, impaired cough reflex, esophageal sphincter relaxation, and reduced laryngopharyngeal sensitivity. These factors have shifted the high-risk group toward this population (13, 14). Interestingly, recent research has shown an increase in iatrogenic causes of aspiration, including intubation, tracheostomy tube changes, dental procedures, and CE. Although few patients reported difficulty swallowing, the majority reported coughing, as well as symptoms such as transient choking, a foreign body sensation, and dyspnea, which varied based on the position of the capsule in the airway. A multicenter study revealed that the right bronchial tree, particularly the right bronchus intermedius and right lower lobe basal segment, is the most common site for capsule aspiration (15). In addition, insights from the study by Ulas et al. indicate that foreign body aspiration (FBA) presents significant diagnostic challenges, especially in children, where non-opaque foreign bodies complicate radiological evaluations and may lead to delays in diagnosis. Their findings suggest that immediate interventions, such as rigid bronchoscopy, are crucial for managing these cases (16).

In terms of aspiration treatment, in some cases, it can be resolved through active cough, chest percussion therapy, and postural drainage. However, CE aspiration often warrants an invasive procedure, with bronchoscopic retrieval preferred to avoid long-term sequelae. Previous bronchoscopic techniques for CE removal included rigid and flexible bronchoscopy, with assistant devices for manipulation, such as foreign body forceps, expandable basket foreign body retrieval devices, and balloon catheters. From the present case and previous case reports, we summarized several experiences and suggestions for improvement, as follows: ① when conducting CE, gastroenterologists should raise their awareness of the above-mentioned high-risk population. All medical histories should be evaluated, especially concerning neurological disorders, degenerative conditions, and swallowing difficulties. To timely localize the capsule, they must pay attention to the endoscopic return image in real time and, if necessary, perform additional auxiliary examinations, such as chest X-ray or CT scan. Next, while managing patients at risk of aspiration, selecting a suitable postural position and providing instructions to modify swallowing techniques are useful. ② Due to the large size and smooth surface of the capsule, the retrieval process is prone to slippage, making the timing of the procedure unpredictable. Therefore, it is recommended to use general anesthesia with the assistance of a laryngeal mask airway for ventilation. ③ As there is no grip point on the surface of capsule endoscopes, using a snare or stone retrieval basket is more advantageous for the retrieval process.④ When aspiration has already occurred and the patient is under general anesthesia, it is recommended that the capsule be directly delivered into the duodenum with gastroscope assistance (17, 18) during the procedure to avoid further aspiration due to active swallowing.⑤ During the process of pushing the capsule into the gastrointestinal tract, it is difficult to accurately guide a gastroscope into the esophagus because the capsule in front of the endoscope blocks the view of its progression. Therefore, adopting a position with the neck flexed and the head bowed or performing tracheal intubation can help prevent the capsule from falling back into the trachea. ⑥ Flexible bronchoscopy should be attempted first in cases where the patient is not in respiratory distress as it carries lower risks and has a high success rate of approximately 90% for foreign body removal. If necessary, early conversion to rigid bronchoscopy is crucial to avoid complications from excessive manipulation. Finally, a surgical thread can be wrapped around the capsule to facilitate emergency retrieval in high-risk patients. ⑦ For manufacturers, it would be beneficial to incorporate a grip point on the surface of the capsule, such as carving a groove in the middle or designing a textured surface that aligns with commonly used retrieval tools, such as snare loops. This would allow for easier and more secure retrieval in cases of aspiration. In addition, modifying the capsule’s size and weight could help reduce the risk of it becoming lodged in the airway. Exploring biocompatible materials with both flexibility and firmness would also enhance the capsule’s maneuverability during its passage through the esophagus and digestive tract, potentially minimizing the risk of accidental aspiration. Another design consideration could be incorporating a retractable safety mechanism or sheath to prevent the capsule from entering the airway during swallowing, further reducing the risk of aspiration in high-risk patients.

The cases detailed above involve elderly male patients who, after inadvertent ingestion, presented with typical symptoms such as persistent cough and chest discomfort. In line with the patterns observed in the majority of cases documented in the literature, the capsule became lodged in the right main bronchus. CE aspiration is specific to the medical device used for gastrointestinal examination, primarily affecting patients with predisposing factors. Its prevention relies on a thorough risk assessment before the procedure. To address this, we opted for the conventional approach of using a flexible bronchoscope for the extraction of the capsule. Subsequently, with the assistance of a gastroscope, we carefully maneuvered the capsule directly into the duodenum, thereby mitigating the potential risk of recurrent aspiration. Our intention in presenting these cases is to raise the awareness of internal medicine practitioners regarding the potential complications associated with capsule aspiration. By sharing our experiences, we aimed to provide valuable insights and serve as a reliable reference for effectively managing this serious complication. It is our fervent hope that this report will prove beneficial in guiding medical professionals on the nuances of dealing with capsule aspiration and underscore the importance of preventive measures to minimize the associated risks.

## Conclusion

In conclusion, more information is needed to tackle aspiration during CE practices. Elderly patients and those with neurological or swallowing disorders and spinal abnormalities should be regarded as a high-risk population for CE aspiration. Performing routine investigations of the related medical history, taking precautionary measures, and monitoring in real-time is essential. Bronchoscopy stands out for its dual functionality, excelling in both meticulous inspection and effective treatment, making it the preferred method for the retrieval of capsule endoscopes. This multifaceted capability not only facilitates successful capsule extraction but also allows for immediate intervention if any complications arise during the procedure. As we navigate the evolving landscape of medical technology, we look forward to advancements from manufacturers in developing capsule endoscopes that are not only scientifically advanced but also equipped with enhanced safety features. By fostering continuous innovation in this field, we can ensure the development of more reliable and secure capsule endoscopy devices for the benefit of both clinicians and patients.

## Data Availability

The original contributions presented in the study are included in the article/supplementary material, further inquiries can be directed to the corresponding author.
